# Marine Sponge/H_3_PO_4_: As a Naturally Occurring Chiral Catalyst for Solvent-free Fischer-Indole Synthesis

**DOI:** 10.17795/jjnpp-11804

**Published:** 2013-11-01

**Authors:** Mohammad Reza Shushizadeh, Azar Mostoufi, Rashid Badri, Somaye Azizyan

**Affiliations:** 1 Department of Medicinal Chemistry, Ahvaz Jundishahpur University of Medical Sciences, Ahvaz, IR Iran; 2 Marine Pharmaceutical Science Research Center, Ahvaz Jundishapur University of Medical Sciences, IR Iran; 3 Islamic Azad University-Khoozestan Science and Research Center, Ahvaz, IR Iran

**Keywords:** Ketones, Indole, Phenylhydrazine

## Abstract

**Background:**

A new and efficient method have been developed for the synthesis of different indole derivatives from various ketones, having at least one hydrogen atom attached to each of their α-carbon atoms, and hydrazines in solvent-free conditions, using marine sponge/H_3_PO_4_ as a naturally occurring chiral catalyst.

**Objectives:**

This study recommended the use of marine sponge/H_3_PO_4_ as a naturally occurring chiral catalyst for preparation of phenylhydrazones from ketones having one α-hydrogen and subsequent cyclisation of the products to indoles.

**Materials and Methods:**

The reaction was carried out by mixing the phenylhydrazine, ketone, and marine sponge/H_3_PO_4_ powder in mortar and pestle; the mixture was ground at room temperature in an appropriate time until TLC show the completion of the reaction. The product extracted by CH_2_Cl_2_ and evaporation of solvent yields the products.

**Results:**

In this research work, several indoles are synthesized using phenylhydrazine and aliphatic or aromatic ketone as starting materials, in the presence of marine sponge/H_3_PO_4_ powder as a natural catalyst under solvent-free condition.

**Conclusions:**

We found marine sponge/H_3_PO_4_ to be an effective catalyst for indolisation of phenylhydrazones from ketones having α-hydrogens in solvent-free conditions.

## 1. Background 

Indole ring systems became important structural components in many natural pharmaceutical agents (1). Their synthesis and functionalization are a major area of focus for synthetic organic chemists. Numerous methods have been developed for the synthesis of indoles (2, 3). The Fischer-indole synthesis, which uses ketones and arylhydrazines is the most widely employed synthetic procedure, especially for large- scale productions of biologically active compounds. This procedure involves an acid- catalyzed 3,3-sigmatropic rearrangement of an N-arylhydrazone intermediate, followed by elimination of ammonia. Several Bronsted acids (H_2_SO_4_, HCl, PPA, AcOH, TsOH, and ionic liquids), Lewis acids (ZnCl_2_, TiCl_4_, BiNO_3_, and Sc(OTf)_3_), and solid acids (zeolite, and Montmorillonite clay) have been reported to catalyze the Fischer-indole synthesis (4-19). Recently, propyl phosphonic acid in combination with cyclic anhydride (T3P) has also been reported recently to catalyze this synthesis (20). Although these methods are satisfactory for the synthesis of many molecules and other purposes, they have certain disadvantages, such as long time reaction, harsh reaction, using high amount of acid catalyst, low products, and using toxic, corrosive, expensive, or non-reusable catalysts. Another major drawback is that these methods do not exhibit any enantioselectivities induced by chiral and natural catalysts. These disadvantages limit their practical utility in large- scale synthesis processes. Consequently, there is a need to develop alternative reagents for these types of reactions. Marine sponges are known as a prolific source of biologically active and structurally unique metabolites (21, 22). We described the catalytic, chirality and absorbent abilities of marine sponge powder of Iranian coast of Persian Gulf in organic reaction such as sulfonamides synthesis (23), and researches about its application in organic reactions such as various oxidation, and reduction reactions (24); therefore we decided to study shallow sponges (*Desmospongea* sp.) of Qeshm and Bushehr Islands in offshore zone as an efficient chiral catalyst.

Other advantages of the marine sponge are that it can act as an absorbent to activate the C–N bond for nucleophilic preparation and cyclisation of phenylhydrazone, with high and predictable asymmetric induction, and can be removed easily from the product. For better catalyst, with respect to operational simplicity, greater yields, indolisation reactions of cyclic and acyclic ketones more research in this field is demanded.

## 2. Objectives

**Figure 1.**
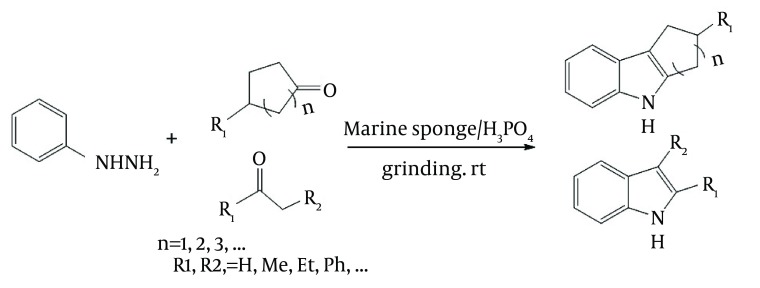
Preparation of Indoles by Cyclization of Hydrazones in Presence of Marine Sponge/H_3_PO_4_

In continuation of our studies on the solvent-free and marine natural catalytic reactions, it was decided to report the use of marine sponge/H_3_PO_4_ as a naturally occurring chiral catalyst for preparation of phenylhydrazones from ketones having one α-hydrogen and subsequent cyclisation of the products to indoles, as shown in Figure 1.

## 3. Materials and Methods 

### 3.1. Reagents and Materials

All starting materials were purchased from Merck and Aldrich Companies. The IR spectra were recorded on a Perkin-Elmer RXI infrared spectrometer. ^1^H NMR spectra were recorded on a 400 MHz Brucker FT-NMR spectrometer. The SEM image was recorded on 1455 VP LEO-Germany. TLC accomplished the purity of substrates and reactions monitored on silica gel (Merck, Germany) polygram SIGL/UV254 plates. The melting points are uncorrected.

### 3.2. Preparation of Marine Sponge Powder

**Figure 2.**
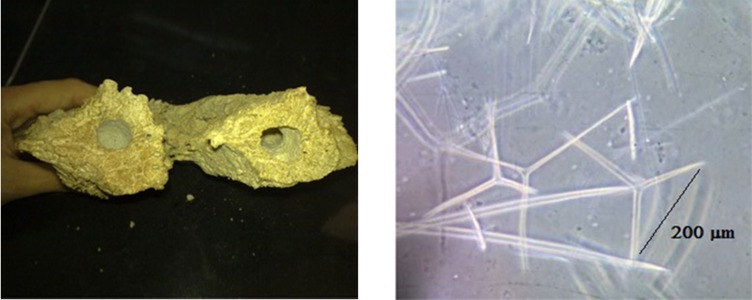
A. Marine Sponge (*Demospongiae* sp.), B. SEM of Siliceous Spicules

In this study, marine sponges (*Demospongiae *sp.) collected from Nakhiloo Island, Bushehr, Iran (North coast of Persian Gulf), in May 2010 at a depth between 5 and 10 m, and were washed several times using methanol and then deionized water to remove some organic compounds, extraneous and salts. Then, they were dried in an oven at 60˚C for 48 hours. The dried marine sponge was chopped and the particles were separated according to their sieves. Identification of sponges was carried out kindly by Khoramshahr marine science and Technology University. It was indicated as *Demospongiae* sp., that has siliceous (SiO_2_) spicules, as shown in Figure 2.

### 3.3. Preparation of Marine Sponge/H3PO4 Mixture

Marine sponge/H_3_PO_4_ mixture was prepared simply by grinding 0.9 g marine sponge powder, and 0.1 mL concentrated phosphoric acid in a mortar until a fine and homogeneous mixture was obtained.

### 3.4. General Procedure

The reaction was carried out by mixing the phenylhydrazine (50 mmol), ketone (50 mmol), and 0.1 g of marine sponge/H_3_PO_4_ mixture in mortar and pestle; the excessive amount of ketones such as acetone and ethyl methyl ketone with low boiling point, was used. The mixture was ground at room temperature in appropriate time reported in Table 1. The progress of reaction was monitored by TLC. After completion of the reaction, the mixture was added to 25 mL of ice/water and stirred. The product was extracted with CH_2_Cl_2_ (2 × 25 mL), and washed with 10 mL of 5% sodium hydrogen carbonate and 10 mL of water. Then the solution was dried over anhydrous CaCl_2_ and filtered. The solvent was evaporated under reduced pressure, and the crude product was purified by recrystallization in 50% ethanol-water to make the pure product. Note: phenylhydrazine is a suspected carcinogen; therefore, gloves should be worn whenever working with compound. Some products are readily oxidized, so they must not be dried in an oven.

**Table 1.**
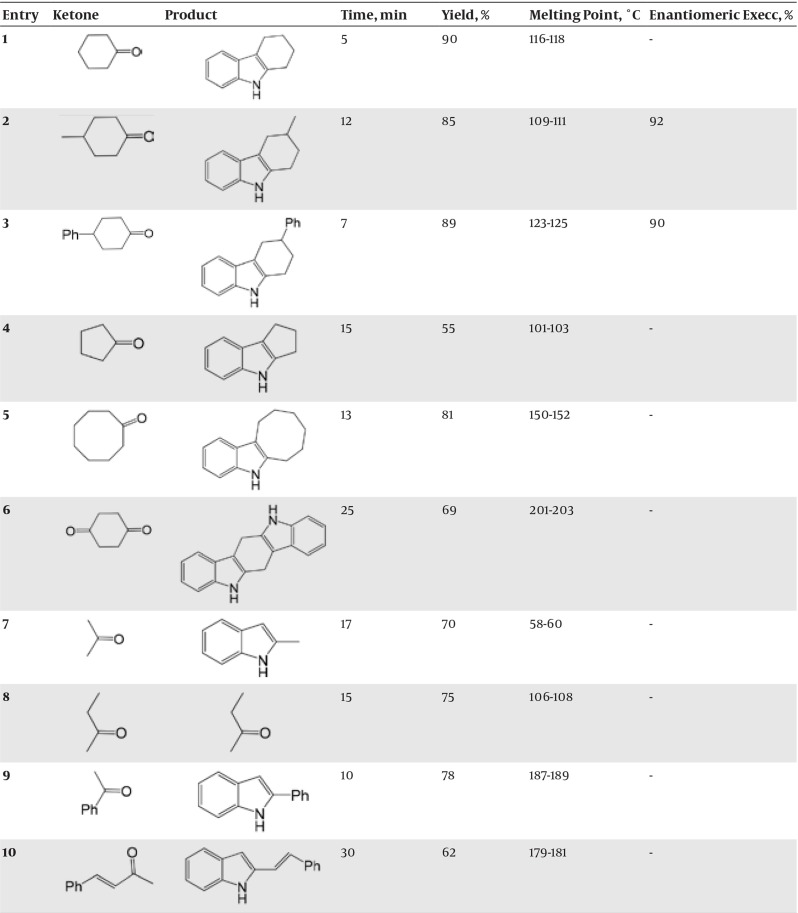
Solvent-Free Fischer-Indole Synthesis of Phenyl-Hydrazine and Ketones Using Marine Sponge/H_3_PO_4_^a^ ^a^ All products were confirmed by comparison with authentic samples (IR, ^1^H NMR and TLC). The reaction was carried out by mixing the phenylhydrazine (50 mmol), ketone (50 mmol), and 0.1 g of marine sponge/H_3_PO_4_ mixture; the excessive amount of ketones such as acetone and ethyl methyl ketone with low boiling point, was used.

## 4. Results

**Figure 3.**
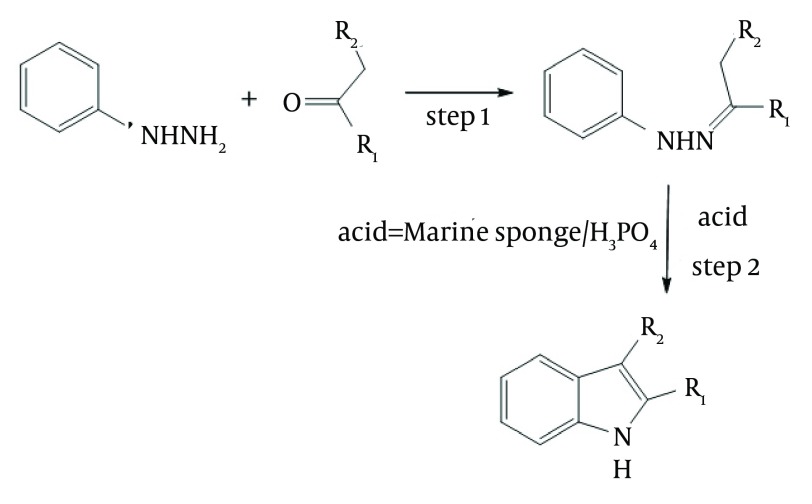
Mechanism of Indoles Synthesis in Presence of Marine Sponge/H_3_PO_4_

Indoles bearing various substituents at positions 2 and 3 can be synthesized via the Fisher-indole synthesis, which involves two steps and uses phenylhydrazine and an aliphatic or aromatic ketone as starting materials. During the synthesis in our experiments, phenylhydrazine reacted with ketone to produce a phenylhydrazone, as shown in step 1 of Figure 3. An acid was added to catalyses, the cyclization reaction and the subsequent loss of one of the nitrogen atoms as ammonia, as shown in step 2 of the Figure 3 (25, 26). Various acid have been used for catalyzing the second reaction.

In order to find out the most effective catalyst for indolisation, we performed the Fischer- indole reaction of 1:1 equimolar phenylhydrazine with ketones at room temperature using various catalysts, as shown in Table 2.

**Table 2.** Solvent-free Fischer-indole Reaction of Phenylhydrazine With Cyclohexanone as Ketone, in the Presence of Different Catalysts

Catalysts	Time, min	Yield, %	Condition
**Marine sponge/H_3_PO_4_**	5	90	r.t.
**[cmmim] [BF4] (12)**	120	92	140˚C
**Propylphosphonic Anhydride (20)**	5	90	MW, 100˚C
**PPA (16)**	15	89	95˚C

## 5. Discussion

As the results indicate, marine sponge/H_3_PO_4_ are more advantageous over previously used catalysts, which are as follows: requirement of a very small amount of the catalyst, mild reaction, room temperature, good solid absorbent , easy to handle, and products in good-to-high yields. More importantly, this catalyst produced enantioselective products with high enantiomeric excess, as shown in Table 1 (entries 2 and 3). Structures of the products were characterized by their spectral (^1^H NMR, IR, and MS) data (27, 28). 

### 5.1. Characterization of Products

Selected spectral data for the products in Table 1 are given:

6,7,8,9-tetrahydro-5H-carbazole (14-15)-(entry 1): IR(cm^-1^, KBr): 3396 (-NH), 1614, 1583 (C=C), 738, 635 (aromatic), ^1^H NMR (400 MHz, CDCl_3_, TMS, δ ppm): 7.67 (1H, bs, NH), 7.55 (1H, d, J=7.52), 7.41(1H, d, J=7.91), 7.22 (1H, t, J=7.75), 7.05 (1H, t, J=7.1), 2.9 (2H, t, CH2, J=3.1), 2.7 (2H, t, CH_2_, J=5.2), 1.8 (4H, m, 2CH_2_); ^13^C NMR (400 MHz, CDCl_3_, TMS, δ ppm): 136.1, 134.1, 127.7, 121.1, 119.0, 117.7, 110.3, 109.0, 24.7, 22.9, 22.0, 21.2.

3-Methyl-2,3,4,9-Tetrahydro-1H-carbazole-(entry 2): IR(cm^-1^, KBr): 3396 (-NH), 1619, 1583 (C=C), 740, 635 (aromatic), ^1^H NMR (400 MHz, CDCl_3_, TMS, δ ppm): 7.6 (1H, bs, NH), 7.5 (1H, d, J=7.5 Hz), 7.38 (1H, d, J=7.95 Hz), 7.25 (1H, t, J=7.7 Hz), 7.03 (1H, t, J=7.1 Hz), 2.9 (2H, t, CH2, J=9 Hz), 2.7 (2H, d, CH_2_, J=6.5 Hz), 2.1 (2H, m, CH_2_), 1.6 (1H, m), 1.2 (3H, d, CH_3_, J= 6.45 Hz); ^13^C NMR (400 MHz, CDCl_3_, TMS, δ ppm): 135.7, 134.1, 128.8, 124.1, 119.0, 117.7, 111.4, 110.0, 30.9, 29.0, 28.3, 26.2, 21.7.

3-Phenyl-2,3,4,9-Tetrahydro-1H-carbazole-(entry 3): IR(cm^-1^, KBr): 3396 (-NH ), 1644, 1604 (C=C), 749, 682 (aromatic), ^1^H NMR (400 MHz, CDCl_3_, TMS, δ ppm): 7.65 (1H, bs, NH), 7.54 (1H, d, J=7.55 Hz), 7.45 (1H, d, J=8 Hz), 7.36 (1H, t, J=7.7 Hz), 7.30 (1H, t, J=7.0 Hz), 7.2 (2H, t, J= 8.1 Hz), 7.15 (3H, m), 3.0 (2H, d, CH_2_, J=11 Hz), 2.8 (2H, d, CH_2_, J=6.9), 2.2 (2H, m, CH_2_), 1.9 (1H, m); ^13^C NMR (400 MHz, CDCl_3_, TMS, δ ppm): 145.8, 136.0 , 135.7, 132.90, 132.92, 128.9,126.7, 126.8, 124.9, 124.1, 119.0, 117.7, 111.4, 110.0, 38.0, 32.3, 30.3, 26.8.

1,2,3,4-Tetrahydrocyclopenta[b]indole (14-15)-(entry 4): IR(cm^-1^, KBr): 3402 (-NH ), 1600, 1502 (C=C), 749, 693 (aromatic), ^1^H NMR (400 MHz, CDCl_3_, TMS, δ ppm): 7.59 (1H, bs, NH), 7.45 (1H, d, J=7.15 Hz), 7.33 (1H, d, J=7.05 Hz), 7.2 (1H, t, J=7.4 Hz), 7.05 (1H, t, J=7.25 Hz), 3.0 (2H, t, CH_2_, J=7 Hz), 2.6 (2H, t, CH_2_, J=7 Hz), 1.59 (2H, p, CH_2_, J=7 Hz);^13^C NMR (400 MHz, CDCl_3_, TMS, δ ppm): 137.0, 135.6, 125.8, 122.0, 120.8, 120.0, 112.1, 111.0, 26.9, 25.8, 24.9.

6,7,8,9,10,11-Hexahydrocycloocta[b]indole (14-15)-(entry 5): IR(cm^-1^, KBr): 3391 (-NH ), 1619, 1599 (C=C), 743, 698 (aromatic), ^1^H NMR (400 MHz, CDCl_3_, TMS, δ ppm): 7.5 (1H, bs, NH), 7.49 (1H, d, J=7.3 Hz), 7.35 (1H, d, J=7.55 Hz), 7.2 (1H, t, J=7.1 Hz), 7.04 (1H, t, J=7.45 Hz), 2.75-2.85 (4H, d, 2CH_2_), 2.5-2.65 (4H, m, 2CH_2_), 2.1 (4H, m, 2CH_2_); ^13^C NMR (400 MHz, CDCl_3_, TMS, δ ppm): 136.6, 135.1, 129.7, 124.7, 119.0, 117.7, 112.6, 110.4, 28.6, 26.5, 26, 25.9, 25.7, 22.2.

5,6,11,12-Tetrahydro-6,12-diaza-indeno[1,2-b]fluorene-(entry 6): IR(cm^-1^, KBr): 3391 (-NH ), 1614, 1583 (C=C), 741, 697 (aromatic), ^1^H NMR (400 MHz, CDCl_3_, TMS, δ ppm): 7.7 (2H, bs, 2NH), 7.6 (2H, d, J= 8.3 Hz), 7.43 (2H, d, J= 7.8 Hz), 7.03 (2H, t, J= 8.15 Hz), 6.7 (2H, t, J= 7.3 Hz), 4.5 (4H, m, 2CH_2_); ^13^C NMR (400 MHz, CDCl_3_, TMS, δ ppm): 136.1 (2C), 132.4 (2C), 124.4 (2C), 118.9 (2C), 118 (2C), 111.7 (2C), 107.7 (4C), 30.9 (2C).

2-Methylindole (14-15)-(entry 7): IR(cm^-1^, KBr): 3399 (-NH), 1558 (C=C), 738, 691 (aromatic), ^1^H NMR (400 MHz, CDCl_3_, TMS, δ ppm): 7.6 (1H, bs, NH), 7.51 (1H, d, J=7.2 Hz), 7.4 (1H, d, J= 7.6 Hz), 7.23 (1H, t, J= 7.44 Hz), 7.1 (1H, t, J=7.1 Hz), 6.7 (1H, m), 1.9-2.3 (3H, s, CH_3_); ^13^C NMR (400 MHz, CDCl_3_, TMS, δ ppm): 137.1, 136.0, 129.6, 120.9, 120.0, 119.6, 110.2, 100.1, 15.9.

2,3-Dimethylindole (14-15)-(entry 8): IR(cm^-1^, KBr): 3402 (-NH ), 1618, 1558 (C=C), 738, 636 (aromatic), ^1^H NMR (400 MHz, CDCl_3_, TMS, δ ppm): 7.45 (1H, bs, NH), 7.35 (1H, d, J=7.5 Hz), 7.35(1H, d, J=8 Hz), 7.15 (1H, t, J=7.78 ), 7.05 (1H, t, J= 7 Hz ), 2.7 (3H, s, CH_3_), 2.5 (3H, s, CH_3_); ^13^C NMR (400 MHz, CDCl_3_, TMS, δ ppm): 137.1, 129.0, 121.4, 120.1, 118.0, 113.6, 110.2, 106.1, 15.9, 10.1.

2-Phenylindole-(entry 9): IR(cm^-1^, KBr): 3350 (-NH ), 1599 (C=C), 744, 682 (aromatic), ^1^H NMR (400 MHz, CDCl_3_, TMS, δ ppm): 7.9 (1H, bs, NH), 7.65 (1H, d, J=7.5 Hz), 7.5 (1H, d, J=8.2 Hz), 7.41 (1H, t, J=7.4 Hz), 7.3 (1H, t, J=7.05 Hz), 7.25 (3H, m), 7.1 (2H, t, J= 8 Hz), 7 (1H, m); ^13^C NMR (400 MHz, CDCl_3_, TMS, δ ppm): 137.7, 135.2, 132.3, 128.3, 128.2, 128, 127, 124.2, 124.1, 121.5, 119.9, 119.1, 110.1, 100.1.

2-Styrylindole-(entry 10): IR(cm^-1^, KBr): 3345 (-NH ), 1601, 1504 (C=C), 748, 693 (aromatic), ^1^H NMR (400 MHz, CDCl_3_, TMS, δ ppm): 8.7 (1H, bs, NH), 7.8 (1H, d, J=7.6 Hz), 7.5 (1H, d, J=7.33 Hz), 7.42 (1H, t, J=7.53 Hz), 7.32 (1H, t, J=7 Hz), 7.2 (3H, m), 7.1 (2H, t, J= 8.3 Hz), 7 (1H, m), 6.9 (1H, d, J=15 Hz), 6.71 (1H, d, J=18 Hz); ^13^C NMR (400 MHz, CDCl_3_, TMS, δ ppm): 145.3, 141.2, 139.2, 129.3 (2C), 128.4, 128.2 (2C), 128.0, 125.6, 120.2, 113.3, 107.4 (2C).

In conclusion, we found marine sponge/H_3_PO_4_ to be an effective catalyst for indolisation of phenylhydrazones from ketones having α-hydrogens in solvent-free conditions. The quality of indolic products is good. This method offered marked improvement compared to previously reported ones. Its advantages included operational simplicity, low reaction time, and high yields of pure products.
